# Selections and Abstracts

**Published:** 1898-09

**Authors:** 


### SELECTIONS AND ABSTRACTS.

### Treatment of Locomotor Ataxia by Systematic Exercise.*

The greatest advance which has been made in the treatment of organic nervous disease is that gained by the exercise treatment of locomotor ataxia. It is true that this is not the treatment of a disease, but of a symptom. But it is the treatment of that symptom—ataxia—which is the most prominent and most disabling, and its improvement or cure oft enables the bedridden, or those in enforced idleness, to again move among their fellows. The treatment to some extent also relieves other symptoms.

So many therapeutic measures of but little or no avail have been tried or vaunted in the treatment of locomotor ataxia that it is a great satisfaction to find a mode of treatment of such signal value.

This treatment consists essentially in this, that movements, which have become ataxic as the result of disease, are practiced systematically with great care until they are again done in a normal, or nearly normal, manner. This is re-education in co-ordinated movements. Originally, all co-ordinated movements are acquired by long practice. The infant learning to walk, the child to write, the piano-player to play, are illustrations of this fact. The act, at first difficult, as it is practiced over and over again, becomes more and more easy until it becomes automatic, or quasi-automatic.

In locomotor ataxia, as the result of loss of sensory† guidance, this acquired co-ordination of movement becomes impaired or lost, and it now needs greater effort to reacquire it than in the beginning. To attain this we must, firstly, call upon what sensory control still remains, and by our strenuous efforts we probably stir into action sensory fibers which have, hitherto, lain dormant. Secondly, we call in the assistance of vision. Its power to substitute, in part, lost sensory guidance we have always seen, in that the ataxic walks better by day than by night, and stands more firmly and uses his hands more skillfully with the eyes opened than closed. Thirdly, the patient must pay the most careful attention

*By Philip Zenner, A.M., M.D., Cincinnati, Lecturer on Nervous Diseases in the Medical College of Ohio. Read by title before the American Medical Association, June 9, 1898.

† The theory of sensory origin of ataxia, though not clearly worked out, nor universally accepted, is constantly gaining in strength.

that the desired movement is made with the greatest possible precision. By means of these three, the remaining, and perhaps to be increased, sensory control, vision, and painstaking effort, and at the same time systematic practice of the given exercises, co-ordination can be reacquired, and ataxia proximately or altogether cured.

The value of this treatment in locomotor ataxia rests upon two facts: Firstly, as any given ataxic movement can be remedied by re-education in co-ordination of given muscles, so all the ataxia manifested may be removed if the proper exercises are given; and, secondly, that locomotor ataxia is usually slow in its progress, often, apparently, stationary.

We owe this treatment to Frenkel, of Heiden, Switzerland. A patient of his at the first examination showed a good deal of ataxia of the hands in trying to bring the index-fingers together, while at the second examination he succeeded in making this test perfectly. The patient stated, in explanation of the great improvement, that he had been constantly practicing the exercise since his first visit. It was after this observation that the thought of systematic treatment of this kind occurred to Frenkel, and he soon demonstrated by actual experience the value of such treatment. Frenkel's first publication was in 1889. Since then he has had abundant opportunity for the application and amplification of his method. The fame of his treatment, and his brilliant results, caused ataxics to flock to his institution in no small numbers, so that his experience and opportunities for observation have been very large. He learned, thereby, that the treatment was applicable not only to light cases, as he had at first supposed, but to very bad cases also, and that the improvement and cure were of indefinite duration. But he learned also that the treatment was not an easy, but a very difficult one, requiring for its success the most careful study of its mode of application to the individual case. So great has been his success that he believes that failures, unless there be contraindications to the treatment, are always due to faulty methods.

Because this treatment is not nearly so well known as its deserts merit, and, at the same time, should belong to the general practitioner, and because my personal experience has taught me how easily failures may result without full knowledge of the rules governing the application of this method, I have deemed the subject well worthy to be brought before the American Medical Association.

To get a clearer idea of what can be done, let us first consider the limitations of treatment.

As contraindications of treatment may be mentioned:

(*a*) Fragility of the bones.

(*b*) Joint disease.

(*c*) Blindness. Vision is essential to the treatment.

(*d*) Mental disease. Nothing can be done without the intelligent co-operation of the patient.

(*e*) A meningeal type of disease, or that with strong cord or root irritation symptoms. Here exercise is likely to increase the pain.

(*f*) Acute, or rapidly progressive disease. Rest, not exercise, is here indicated.

(*g*) Spasticity or muscular atrophy, especially if of high degree.

(*h*) Serious organic disease, especially heart disease. If the conditions still permit the treatment, it should be instituted with great care.

Other conditions require preparation for the treatment. In case of anemia, or bad nutrition, or other impairment of health, the general health should be restored before beginning the exercise treatment. Frenkel has called special attention to a condition of the muscles in locomotor ataxia, which he terms hypotonus, a loss of muscular tone, making the joints less firm, and excessive movement possible. For instance, a normal individual lying on his back can usually lift his leg but to a right angle, while with the hypotonus spoken of he can bring the leg much nearer the trunk. This hypotonus, causing relaxed muscles and tendons and weakened joints, must be relieved by electricity, massage, bandages and the like, before much benefit can be expected from the exercise treatment.

The favorable cases for this treatment are those in good general health, intelligent and hopeful, where the disease is advancing very slowly, or is, practically, at a standstill. The lighter forms are the more easily and quickly improved, but bad cases, though the treatment is necessarily of longer duration, often give brilliant results.

Let us for a moment consider the different forms or degrees of ataxia which may be presented.

The lightest and usually first to appear, is static ataxia. The patient sways when standing with closed eyes, is unsteady if he stands on one foot, cannot stand on tip toes, etc. A step further and locomotion becomes difficult; the gait is taxic, first manifested in running, dancing, sudden turning and the like. A still higher degree of ataxia is that of the separate limbs. Even in a position of rest the ataxia of the leg may be shown in the unsteady way the patient touches the knee of one leg with the heel of the other foot, or like movements; and the same may be seen in the movements of the hands, if the upper extremities be affected. Still higher degrees of ataxia are those where the patient can hardly lift himself from the chair, needs support on one or both sides in walking or where any movement becomes difficult or almost impossible. The bedridden, so-called paralytic, cases are often only cases of extreme loss of coordinating power, proven by their improvement under this method of treatment.

For these various degrees and location of ataxia differences in the method of treatment are called for, as to the choice of exercises, frequency and duration of treatment, etc.

I give herewith a series of exercises described by Hirschberg,* who supervised their execution under Frenkel's instruction at the Salpetriere.

EXERCISES FOR THE TRUNK AND LOWER EXTREMITIES.

*Exercises in Bed.*—The patient is laid on a bed or sofa, the legs bare, the head raised so he can see his legs; then he performs the following exercises:

1. Flexion, extension, abduction and adduction of one foot, then the other, then both together.
2. Movement of rotation at ankle.
3. Flexion of knee.
4. Flex leg on abdomen with knee bent.
5. Abduction and adduction of thigh, knee bent, sole of foot to rest on bed, pelvis immobile; to be done in four stages—abduction, return to median line; adduction, return to median line.
6. Raise the leg as a whole, without making zigzag movements; then practice abduction and adduction and rotation with the leg at the hip-joint.
7. The legs are straightened out and brought together; the patient is then to raise himself in bed without the use of hands and without moving his legs.

*Standing Exercises.*—For these exercises one should have a large, well-lit room with little furniture, with a floor which has not been waxed or covered with a carpet. The patient ought to be lightly clothed. Women should wear bloomers. At the beginning of treatment it is very important that patients should be able to see their legs.

*Exercises in Static Equilibrium.*—The patient is standing, the doctor at his side. If the patient cannot walk he is to be supported by the aid of Frenkel's belt. If he can hold himself erect by the aid of a cane this is permitted him in the outset.

1. Stand immobile, the feet slightly separated, hands resting at the side. Stand in this position one or two minutes.

*Archives de Neurologie*, 1896, Nos. 9 and 11.

2. Same exercise, feet close together.
3. With the feet a little apart, do exercises with the arms; raise them, lower them, extend them, etc.
4. Same exercises with the feet close together.
5. Bend body in different directions, moving head also.
6. Same exercises with feet together.
7. Bend forward and straighten up slowly.
8. Same with feet together.
9. Bend body forward and touch toes with tips of fingers.
10. Same with feet together.
11. Stand on toes.
12. Same with feet together.
13. Stand with knees bent.
14. Same with feet together.
15. While standing with knees bent exercise arms.
16. Stand on one leg.
17. While standing on one leg bend the knee.

EXERCISES OF LOCOMOTION.

1. While erect extend foot slowly length of one step; bring back foot into place with one movement; repeat this, putting foot backwards. Do this sideways. For this exercise it is well to mark on the floor with a piece of chalk just where the foot shall be placed. Perform these exercises with both feet.
2. Put one foot in front of the other in the same line, and stand quietly.
3. Walk forward twenty steps, putting the feet down gently, and touching the floor with the entire foot. The patient should count his steps aloud.
4. Walk along a straight line—a broad stripe painted on the floor.
5. Walk backwards.
6. Walk sideways.
7. Walk with long stretches.
8. Walk with knees bent.
9. Walk on the toes.
10. Walk, at command, with sudden stop or change of direction.
11. Walk with obstacles: pieces of wood are placed on the floor at equal distances from one another, and the patient should walk between them without displacing them.
12. Get up from chair without use of hands.
13. Sit down slowly and carefully.
14. Exercise one's-self in going up and down stairs without holding on to the banister.

For ataxia of the upper extremities very many simple or complex movements may be resorted to—simple movements of fingers, hands and arms, the ordinary test movements by which we commonly test for ataxia, etc. As skilled movements of the hands mean much greater nicety of action than that required of the legs and trunk, Frenkel has devised a number of apparatus for such exercises. For instance, balls of different sizes are suspended by strings; the balls are set in motion, and the patient is requested to grasp a given one between finger and thumb. Again, a board containing a number of holes in which are fitted violin keys or stops is placed before the patient. He is ordered to remove and replace certain specified number stops. He holds the hand behind the head and slowly or rapidly executes the desired movement.*

Dana (*The Post-Graduate*) gives the following exercise for the hands and arms:

1. Sit in front of a table, place the hand upon it, then elevate each finger as far as possible. Then, raising the hand slightly, extend and then flex each finger and thumb as far as possible. Do this first with the right hand and then with the left. Repeat once.

2. With the hand extended on the table, abduct the thumb, and then each finger separately, as far as possible. Repeat three times.

3. Touch with the end of the thumb each finger-tip separately and exactly. Then touch the middle of each phalanx of each of the four fingers with the tip of the thumb. Repeat three times.

4. Place the hand in the position of piano-playing, and elevate the thumb and fingers in succession, bringing them down again, as in striking the notes of the piano. Do this twenty times with the right hand, and same with the left.

5. Sit at table with a large sheet of paper and pencil, make four dots in the four corners of the paper and one in the center. Draw lines from corner dots to center dot with right hand; same with left.

6. Draw another set of lines, parallel to the first, with the right hand; same with left.

7. Throw ten pennies upon the paper, pick them up and place them in a single pile with the right hand; then with the left. Repeat twice.

8. Spread the pennies about on the table, touch each one exactly with the forefinger of right hand; then with the forefinger of left.

9. Place ordinary solitaire board on the table, with the marbles in the grooves around the holes. Put the marbles in their places with right hand; same with left hand. Patient may with advantage practice the game for the purpose of steadying his hand.

*Bettman, *Journal American Medical Association*, January 2, 1897.

10. Take ordinary fox-and-geese board, with holes and pegs, and, beginning at one corner, place the pegs in the holes, one after the other, using first the right hand, then the left.

The physician should select from this long array of exercises those most appropriate for his case, or he may find others still more suitable. The idea is an exercise at first performed in an ataxic manner is done over and over again until it is executed in a normal, or nearly normal, manner. Usually the exercises are done for a half hour or an hour once or twice a day, with rest pauses between the different acts. In this, too, one must individualize. Bad bedridden cases should at first not attempt much, perhaps exercise a very little every hour.

Frenkel has his patients exercise in the morning in bed. These bed exercises he terms the *a b c* of the coordinating act of the lower extremities, and he permits cases with light degrees of ataxia to omit them altogether. Then in the course of the day his patient practices in sitting, standing, walking, promenading, climbing steps, etc., according to the character of the case.

Frenkel emphasizes very strongly the danger from over-fatigue in these cases. It must be remembered, if these exercises are properly done, the patient making most strenuous efforts to do them with exact precision, they tax him both mentally and physically. At the same time the sense of fatigue is usually more or less blunted in ataxics, so that the first intimation of fatigue or over-fatigue may be the increased ataxia which the undue exertion has caused.

For this reason one must constantly guard his patient from over-fatigue. The exercises should not be too prolonged nor such as are too taxing for him. Frenkel is accustomed to have the physician at the side of the patient when he is exercising in standing and walking, so as to save him the added effort or strain that would accrue if he were constantly fearing or trying to prevent himself from falling. Then the patient should not waste his time and strength on unnecessary exercises, those that he already does easily and well, repeated simple muscular contractions not related to his ataxia, etc. It must not be forgotten that the exercises are not for the purpose of increasing muscle strength, but to remove ataxia.

Frenkel denounces severely the use of ordinary gymnastics in these cases. He even warns those in the pre-ataxia stage of tabes against over-fatigue; and advises no excessive walking, no bicycling, no horseback riding, etc. In such cases Frenkel is also accustomed to have massage—muscle-kneading, not skin-rubbing—applied to all his patients for the purpose of improving the muscle tone, and, possibly lessening the effects of fatigue.

The necessary duration of treatment varies according to the severity of the case, from a month or more for the light cases to six months or a year for the bad ones. But the exercises must be continued permanently to maintain the improvement. Frenkel has cases under observation in which the improvement or apparent cure has already been of several years' duration. In case of exacerbation of the disease the improvement in the ataxia gained by the treatment is temporarily lost, but it is easily regained by the same treatment after the relapse is over.

The improvement depends much on the temperament and intelligence of the patient. The nervous, restless, those with little power of attention and weak will power, often make little progress. Occasionally improvement is very slow without any known cause, though, as already mentioned, Frenkel thinks that failure, when there is no contraindication, is always due to faulty methods. He says the latter especially lead to disappointment in those quite common cases where a degree of improvement is easily and quickly attained, while further progress is always slow, and only to be obtained when the method of treatment is thoroughly understood.

This treatment, as already stated, is that of a symptom, and has no effect whatever upon the structural disease. But while it is intended only for the relief of ataxia, other symptoms have sometimes been benefited, notably the lancinating pains and crises of tabes. The spinal myosis and Westphal symptom—lost knee jerks—are unaffected by the treatment.

Frenkel speaks of the ofttimes necessity of institutional treatment, in order to obtain benefit from the treatment, or at least the largest possible benefit. There is no doubt that one who has had Frenkel's large experience in this treatment is best prepared to apply it to the individual case, nor that a patient in Frenkel's institution who sees the brilliant results there obtained would be so buoyed up with hope and the expectation of a cure as to put new life into him and a vigor into his efforts not otherwise attainable. But not every case of locomotor ataxia can go to Heiden or Paris, and there is no reason why the general practitioner, if he studies the method of treatment, and studies his patient, should not gain very much from the treatment in suitable cases. But unless he carefully supervises the treatment he can expect nothing but failure.

I shall attempt to formulate what has been said in this paper in the following rules:

1. All cases should be benefited by the exercise treatment, many to the degree of apparent recovery, unless there be special contraindication to the treatment. Failures under these circumstances usually mean faulty methods, or that the treatment has not been persevered in sufficiently long.

2. Contraindications are: Loss of vision, mental impairment, bone and joint disease, spasticity and muscular atrophy, the presence of strong irritation symptoms, rapid progress of the disease, a state of great exhaustibility, and serious organic disease.

3. In cases of anemia, poor nutrition and lax joints, these general and local conditions should be remedied before the treatment is instituted.

4. The conditions most favorable for the treatment are, a stationary, or almost stationary, state of the disease; good general health, intelligence, hopefulness, and perseverance.

5. Light cases are more amenable to a (practical) cure, but bad, even bedridden, cases often give brilliant results.

6. The necessary duration of treatment varies from a month or more for the lightest, to six months or a year for bad cases; but the exercises must be kept up in order to insure the continuance of the improvement.

7. Success of treatment depends upon thorough knowledge of the method. This is especially true of bad cases.

8. Exercise should be chosen most suitable to remedy the existing ataxia, and every effort should be made to do them with greatest precision.

9. The sense of fatigue is often blunted in ataxics, while over-fatigue injures them. The patient should therefore be guarded against too taxing or too prolonged exercises or other unnecessary efforts.

10. To obtain most benefit from the treatment the constant supervision of the physician, at least in its early periods, is absolutely necessary.—*Cincinnati Lancet-Clinic.*

---

### THE BEARING OF PATHOLOGICAL PROCESSES ON THE THERAPY OF MORBID CONDITIONS ALONG THE GENITO-URINARY TRACT IN THE MALE.

Thomas H. Manley, M.D., Professor of Surgery in the New York Clinical School of Medicine, contributes a paper with this title to *The Canadian Medical Journal*, June, 1898, in which he says:

"In no larger classes of maladies common to the human subject has the rate of progress been greater in the diagnosis and treatment of them during the past twenty-five years than those involving the genito-urinary organs.

"The tendency to mutilation and severe mechanical interference has, no doubt, very often been too great. In the management of calculus of the renal pelvis, the ureter, the bladder, or urethra, the employment of the crusher or blade is yet imperative, but in tuberculosis, prostatic disease or cystic inflammation, the tendency now is in the direction of reaction and less severe surgical measures. This is the attitude of the French school, as expressed by Guyon.

"Tuberculosis of the urinary tract, or the kidney, was a condition but imperfectly understood until of late years. But now we know that, exclusive of blennorrhagic infection, there is probably no pyogenic microbe so prolific as a factor in renal suppuration as the bacillus of tuberculosis. When a knowledge of this fact came into our possession, it was assumed that in its treatment the same principles must apply as with the management of a tubercular lesion elsewhere, viz., by an early ablation of the focus involved, even nephrectomy, when the entire kidney was involved. But events have transpired which have turned us around, so to speak. The mortality has been very large after operations, and, further, we had no assurance that the other kidney was not involved. One might say, however, that any one was a bungler and behind the times who did not determine beyond peradventure by ureteral catheterization whether one or both kidneys were involved, by pyogenic processes.

"It may be well to remember, in this connection, in spite of Alberan's, Kelly's, or Neisser's cystoscopic devices, ureteral catheterization in the male is impracticable in any other than exceptionable cases. This was so declared at a late meeting of Genito-urinary Section of the Academy of Medicine in New York. We have, further, learned the salutary lesson that certain types of renal tuberculosis are frequently amenable and curable by simple and safe expedients.

"My own experience has been that when renal tuberculosis develops, consecutive to pulmonary invasion, the progress of the disease toward death is rapid. On the contrary, when the disease is unilateral or ascending, appropriate treatment is rewarded by gratifying results.

"In the beginning it may be well to bear in mind that when tubercular destruction seizes on any epithelial structure its behavior is quite the same after the stages of vascular stasis and inflammation, suppuration and ulceration set in. Now, in the lung, while the vomica is forming and the residual putrid elements of inflammation and decomposition are accumulating, the constitutional disturbances and local distress are very great; but let them burst into a bronchus or out through the chest walls, immediate relief follows. The cavity again filling without a free escape of its contents, we have *reinfection* and great misery; not from local affection so much as from misery incurred through an ascending infection of the bronchi, the tracheal and laryngeal mucous membrane. In lung affections an insurmountable difficulty comes through the carrying away infected products, as we *cannot drain up-hill*, gravity being against us. In renal tuberculosis, on the contrary, the advantage of gravity is with us; and more, once the abscess opens into a uriniferous tubule, an incessant stream of fluid is carrying downward and out of the body its contents.

"The attitude of the body, then, is a most helpful aid in renal drainage. Trouble comes here, nevertheless, as with the pulmonary organs, from stasis and stenosis.

"When the purulent discharge from the kidneys consists of a mixed infection with a predominance of the streptococcus, the mucous membrane of the prostate, the ureter or the bladder becomes involved; for some unknown reason the urethral mucous membrane escapes. The mucous membrane becomes infiltrated and thickened, ulcerated or destroyed in severe cases. When the cystic mucous membrane is involved, inflammatory hyperplasia extends into and through the muscular walls, with the result that the bladder discharges the urine incompletely. A residual quantity remains, decomposition and ammoniacal reaction begins, the purulent drip from the ureter now undergoing a mucoid ropy transformation, a condition always resulting from the action of alkali on pus.

"When this stage is reached the miseries and woes of the afflicted are great. The racking, harassing cough of tubercular bronchitis is trying enough, but the torturing tenesmus and strangury of cystic tuberculosis is a most agonizing state.

"Happily, in the great majority of cases, local and constitutional treatment will yield surprising results, and, in most cases, dispense with the need of radical surgery.

*Prostatic Lesions.*—The prostate is an organ of whose existence we are quite unconscious until it makes its way into the bladder, gives off an outgrowth from its isthmus, and blocks up the urethra.

"About midage it is prone to begin hypertrophic changes, alter its position and undergo neoplastic mutations. These pathological changes in themselves are entirely innocuous, and only become a source of trouble when they invade the bladder; and this they often do to such an degree as to make advanced age miserable.

"Besides, they are not infrequently the cause of death, through retrograde changes, extending up the ureters into the kidneys.

"Our hopes of successfully dealing with the prostate by radical measures have been most disappointing. Prostatectomy, perineal or suprapubic, is full of peril, but few surviving the operation. Castration is a procedure of questionable propriety, if catheter-life is possible. Fortunately, as with renal tuberculosis, very much can be accomplished in these cases by simple measures.

"Let us not overlook the fact that in a large number the enlargement is not neoplastic at all, but simply a vascular turgescence, with probably an admixture of inflammatory deposits; or, in other words, that the condition is transient, and not that it is a *cause* of vesical implication, but that it is a *sequence* of morbid conditions within the bladder itself. The urinary stasis or prostatic enlargement, under many circumstances, is dependent upon a more complex pathology than is generally supposed, and we have reason to believe that the initial factors are *vesical.*

"At the stage of life when this begins, inertia of both smooth and stripped muscles commences.

"The general atheroma of interstitial vascular changes which impair the nutrition of the alimentary canal also begin to tell on the walls of the bladder. The organ fails to completely contract or expel its contents, and hence residual urine remains. Pavy found that transient glycosuria is not uncommon in those past middle life. Here we have a change in the composition of the urine, with the necessary ferment to stir into activity changes of decomposition and bacterial action.

"Cystitis begins, and the infection is promptly propagated into the collar of glandular tissue which is essentially an integral part of the bladder.

"From the foregoing it is therefore apparent that if we would relieve the prostate we must begin with the urethra and the bladder; for in all these cases there is invariably a coincident *deep* urethritis.

"First, washing, irrigating.

"The passage opened, we irrigate, first with an abundance of medicated solution; the carbolized being the most valuable.

"The morphological elements of the urine from day to day will tell us how the case is progressing. And when the urine has cleared up and inflammation has ceased, a cure is effected which is often permanent. In some cases, however, the artificial drain must be employed, and catheterization continued indefinitely."—*Medical Review of Reviews.*

